# Whole-genome sequencing to investigate the prevalence and transmission of multidrug-resistant Gram-negative pathogens in an adult intensive care unit in the UK

**DOI:** 10.1099/mgen.0.001654

**Published:** 2026-05-06

**Authors:** Hayley J. Wilson, Petra Polgarova, Fahad A. Khokhar, Ben Warne, Rory Carpenter, Beth Blane, David A. Enoch, Nicholas M. Brown, Jacobus Preller, Charlotte Summers, Sharon J. Peacock, Gordon Dougan, Julian Parkhill, Nicholas R. Thomson, Mili Estée Török

**Affiliations:** 1 Department of Veterinary Medicine, University of Cambridge, London, UK; 2 Cambridge University Hospitals NHS Foundation Trust, Cambridge, UK; 3 Department of Medicine, University of Cambridge, Cambridge, UK; 4 Clinical Microbiology & Public Health Laboratory, UK Health Security Agency, Cambridge, UK; 5 Centre for Translational Stem Cell Biology, Hong Kong, PR China; 6 Wellcome Sanger Institute, Hinxton, UK

**Keywords:** antimicrobial resistance, carbapenem-resistant Enterobacterales (CRE), extended-spectrum beta-lactamase (ESBL), intensive care unit (ICU), multidrug resistance (MDR), whole-genome sequencing

## Abstract

**Background.** Rates of multidrug resistance (MDR) in Gram-negative bacteria (GNB), particularly those harbouring extended-spectrum beta-lactamases (ESBL) and/or carbapenemases, are increasing globally. Intensive care unit (ICU) patients are vulnerable to healthcare-associated infection (HCAI). Surveillance for carriage of multidrug-resistant Gram-negative bacteria (MDR GNB) is inconsistent, with differing practices amongst ICUs, hospitals and countries. Furthermore, the impact of asymptomatic carriage on HCAI rates is unclear.

**Methods.** We conducted a 6-month prospective surveillance study of MDR GNB in a UK adult ICU. Screening samples were collected from all study participants on admission, once a week (depending on length of stay) and on discharge from the ICU. Whole-genome sequencing (WGS) was used to examine the population structure and antimicrobial resistance mechanisms of MDR GNB and to determine evidence of recent transmission between patients.

**Findings.** Of 424 participants recruited between June and December 2016, 15% (*n*=64) were positive for MDR GNB during admission screening to the ICU. The most frequently identified organisms were *Pseudomonas aeruginosa*, *Escherichia coli* and *Klebsiella pneumoniae*. In total, 10% (*n*=42) of patients acquired an ESBL-producing or carbapenem-resistant MDR GNB during their ICU admission. WGS of the bacterial populations reflected national trends. An undetected outbreak of carbapenemase-producing *K. pneumoniae*, which had spread to several wards, was identified and controlled. Most positive patients carried identical lineages across multiple body sites.

**Interpretation.** Our findings suggest that prospective screening for MDR GNB in ICU patients could be beneficial and be considered in other UK critical care settings. This would not only improve early detection but also enable the prompt institution of enhanced infection control measures.

## Data Summary

All genomic sequence data have been deposited in the European Nucleotide Archive under the respective project identifiers. Sequences new to this project can be found under projects PRJEB20809 and PRJEB14854 with accession numbers for each isolate detailed in Supplementary Table S1. All previously published data can be found publicly using the accession numbers detailed in Table S1. All supporting data, code and protocols have been provided within the article or through supplementary data files.

**Impact Statement**

In the UK, there has been an increase in the prevalence of multidrug-resistant Gram-negative bacterial (MDR GNB) infections over the past decade. Patients requiring complex and demanding medical care are disproportionately affected by these pathogens. Bacterial populations carried asymptomatically undermine efforts to reduce MDR pathogen burden and are not subject to the same mandatory surveillance requirements for Gram-negative bloodstream infections in the UK. The impact of the asymptomatic carriage of MDR GNB on critically ill patients remains unclear.

We conducted a prospective genomic surveillance study for MDR GNB in all patients admitted to an adult ICU over a 6-month period in the UK. Prior to this study, patients admitted to the adult ICU underwent screening for carbapenemase-producing *Enterobacterales* (CPE) only if they had defined risk factors (i.e. travel abroad or admission to other hospitals in the UK with high CPE rates). Here, every patient admitted to the ICU was screened for MDR GNB, regardless of whether they met screening recommendations. We screened patients at multiple sites simultaneously and longitudinally, enabling us to generate a comprehensive picture of MDR GNB carriage and infection in this patient group. Asymptomatic carriage of *E. coli*, *K. pneumoniae* and *P. aeruginosa* was identified in several patients. We also detected an unsuspected outbreak of carbapenem-resistant *K. pneumoniae*, which had spread to several wards. This enabled the Infection Prevention Control Team (IPC) to institute enhanced screening and intensified IPC measures to control the outbreak.

This study identified multiple patients with asymptomatic carriage of MDR GNB. The genomic data supported evidence of recent transmission and potential novel lineages but also indicated that community carriage lineages were introduced into healthcare settings. Transmission was not widespread, indicating that IPC protocols in our hospital are largely effective. However, given that many of these lineages have become endemic in the UK, screening criteria for these pathogens may need to be reassessed.

## Introduction

Multidrug-resistant (MDR) *Enterobacterales* dominate the epidemic of antimicrobial resistance. Their increase, particularly *Klebsiella pneumoniae*, and resistance to all but last-line antimicrobials limit treatment options. Extended-spectrum beta-lactamase-producing *Enterobacterales* (ESBL-E) and carbapenem-resistant *Enterobacterales* (CRE) are major culprits and are included in the World Health Organization’s list of priority pathogens, which require urgent action to mediate the continued spread of resistance [1]. European surveillance data estimated that 800,000 infections in the EU/EEA in 2020 were due to antibiotic-resistant bacteria along with continued large increases in carbapenem resistance in *K. pneumoniae* [2].

Patients in intensive care units (ICUs) are disproportionately affected by multidrug-resistant Gram-negative bacteria (MDR GNB) due to frequent comorbidities, chronic health conditions and high antimicrobial usage [3]. ICU physicians view infections caused by antimicrobial-resistant bacteria as a major (24.2%) or moderate (33.9%) problem [3]. Infections with MDR GNB can be traced to multiple sources including community acquisition, environmental contamination and transmission within healthcare networks. Surveillance in the UK includes mandatory reporting of *Escherichia coli*, *K. pneumoniae* and *Pseudomonas aeruginosa* bloodstream infections and monitoring of carbapenem-resistant (CR) pathogens [4]. The increase in antimicrobial resistance in the ICU has been attributed to high-risk clones of ESKAPE pathogens, particularly *E. coli*, *K. pneumoniae* and *P. aeruginosa* [5]. However, the impact of asymptomatic populations and longitudinal population dynamics has not been fully elucidated. Dissemination of MDR GNB is multifaceted and complex. ESBL and CR mechanisms are frequently located on plasmids, which move between lineages, species and genera. These frequently mobile elements are shared within lineages, sequence types (STs) and species, which complicates tracking antimicrobial resistance (AMR) gene transmission in epidemiological and molecular investigations [6].

Whole-genome sequencing (WGS) is a useful tool for understanding bacterial populations and is regularly used in outbreak management and large-scale phylogenomics [7,9]. A previous study conducted in Vietnamese ICUs found high rates of carriage and transmission of MDR GNBs [10]. The aim of this study was to determine the rates of carriage, infection and transmission of MDR GNB in a UK ICU setting using a genomic epidemiology approach, to inform screening and management strategies.

## Methods

### Study design, setting and participants

**Fig. 1.**
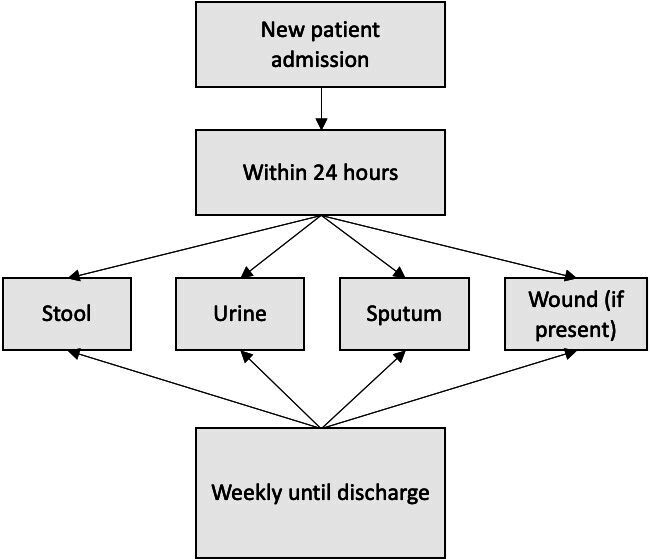
Flow diagram demonstrating the sampling framework in patients.

We conducted a 6-month prospective surveillance study of all adults admitted to the John Farman ICU at Cambridge University Hospitals NHS Foundation Trust (CUH) to determine the rates of carriage, infection and transmission of MDR GNB (defined as Gram-negative bacteria with resistance to three or more classes of antibiotics). The unit was a 20-bedded general adult ICU providing care to medical and surgical patients in the hospital and specialties such as solid organ transplantation, hepatology and haemato-oncology. Patients were admitted from other wards, directly from the Emergency Department, or via other care routes including other hospitals. A floor plan of the unit is provided in Supplementary Data S1 Fig. S1 (available in the online Supplementary Material) for reference. The screening protocol was developed for this study. Prior to this, screening was only carried out in patients known to be positive for MDR GNB or those with a history of travel to a high-risk location. Participants underwent multisite screening for MDR GNB on admission to ICU, weekly during their ICU stay (if the stay lasted 7 days or more) and on discharge from ICU. Screening specimens included a stool sample or rectal swab, a urine sample, a sputum sample or tracheal aspirate and wound swabs (if present) (see Fig. 1).

Patients were deemed to be positive on admission if a positive sample was collected within 24 h of admission to ICU. Patients were deemed to have acquired an MDR GNB if they had any site test positive after previously being negative. Positive samples in a site that had not previously been tested (e.g. missed tracheal sample) were not counted as acquisitions and counted in overall positivity rates. Samples ordered by clinicians in the case of suspected infection were also collected once the clinical laboratory had completed processing them. The distinction between carriage or infection in patients was made based upon the supervising clinician’s decision to treat with antibiotic therapy. If a target organism was isolated from a site where active infection was being treated (e.g. ESBL *E. coli* grown from the urine of a patient being treated for a suspected urinary tract infection), then this was classified as an infection. Any organisms grown from a normally sterile site (e.g. blood cultures, cerebrospinal fluid and joint fluids) were also treated as infection. Clinical, epidemiological and laboratory data were collected from the hospital information system (EPIC; Epic Systems, Wisconsin, USA). Following identification of an outbreak of *K. pneumoniae*, we sought to identify isolates collected elsewhere in the hospital to determine their association with the outbreak. Electronic medical records were examined for all patients with *K. pneumoniae* positive blood cultures between 2016 and 2018. Environmental sampling was performed monthly on the ICU and once on ward 5 (as directed by the hospital IPC team during an outbreak investigation).

**Fig. 2.**
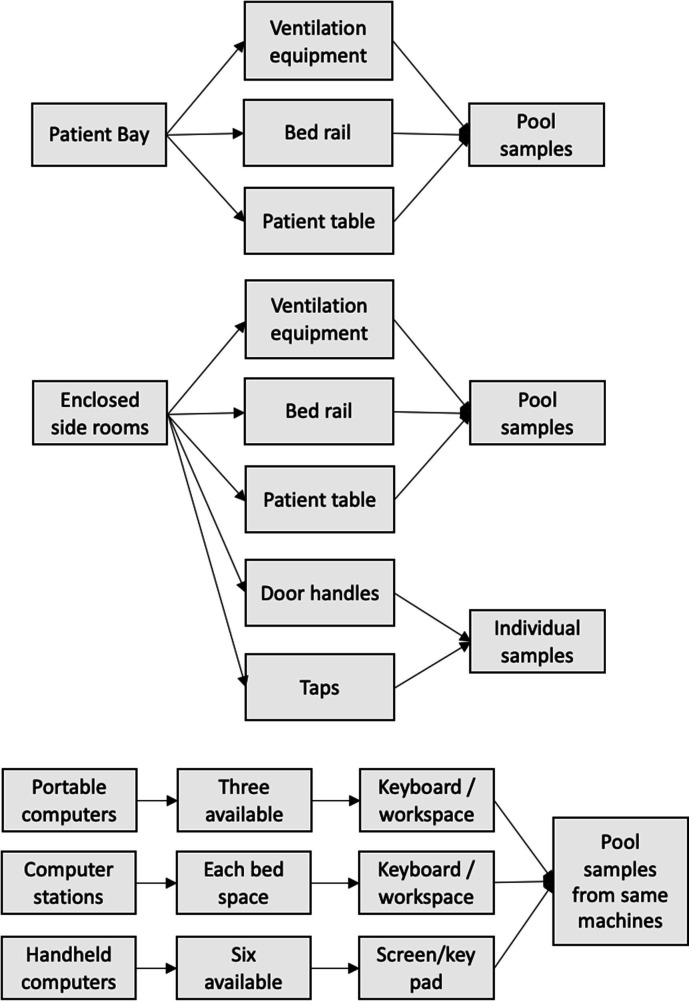
Flow diagram demonstrating the environmental sampling process.

Monthly environmental sampling was performed using flocked swabs (Copan Diagnostics, CA, USA). A 10×10 cm sample was collected from each bed bay or side room, ventilation equipment, bed rails, table, door handles, taps, computer stations and portable computers (see Fig. 2). Samples were collected at a fixed point each month rather than before or after specific patient discharge or cleaning protocols. This was to identify potential pathogens during the routine operation of the ICU.

### Laboratory methods

Specimens were cultured on selective media: chromID CARBA SMART biplates (bioMérieux, Marcy l’Etoile, France) and Brilliance ESBL agar (Oxoid, Basingstoke, UK). Each swab was used to inoculate an agar plate. Ten microlitres of urine, sputum or tracheal aspirate [after pre-treatment with Mucolyse (Prolab Diagnostics, Ontario, Canada)] was plated to the screening media. Ten microlitres of each stool sample was resuspended in 5 ml PBS. Ten microlitres of this suspension was then used to inoculate the screening media. Agar plates were incubated in air at 37 °C for up to 24 h.

Suspect colonies were identified using MALDI-TOF MS (Bruker Daltonics, Bremen, Germany). Antimicrobial susceptibility testing was performed using VITEK^®^2 (bioMérieux). Target organisms (ESBL-E or CR organisms) were stored at −80 °C prior to undergoing DNA extraction and WGS.

### Sequencing and bioinformatic analysis

Genomic DNA was extracted using the QIAcube and the QIAamp 96 DNA QIAcube HT kit (QIAgen, Hilden, Germany), and 150 bp paired-end libraries were prepared according to standard Illumina protocols. Reads were checked for quality using fastQC (https://www.bioinformatics.babraham.ac.uk/projects/fastqc/) and contamination using Kraken [11]. Isolates were assembled using Velvet [12] and VelvetOptimiser (http://www.vicbioinformatics.com/software.velvetoptimiser.shtml). Assembly improvement was completed using assemblies with the best N50 value. Contig scaffolding was performed using SSPACE, and the remaining gaps were closed using GapFiller [13]. Optimized assemblies were annotated using PROKKA v1.11 [14] and a genus-specific RefSeq database.

Core genome phylogenies (1,199 conserved genes) were created using Roary for all *E. coli* study isolates combined with contextual isolates from the UK [15] using Escherichia_coli_ST131_strain_EC958_v1 (GCA_000285655) as the reference and separately all *K. pneumoniae* (2,918 conserved genes) isolates identified in the study using NTUH-K2044 (AP006725) as the reference genome. The reference PA14 was used for *P. aeruginosa* (NCTC01332) (4,105 conserved genes). SAMtools mpileup v0.1 was used to identify variants and bcftools v0.1.19 to produce a Bim Collaboration Fromat (BCF) file of all variant sites. Variant quality scores greater than 50 and mapping quality scores greater than 30 were used. Bases called at each site in the BCF file were substituted into the reference genome to create a pseudo-genome with uncertain bases or deletions in the context of the reference genome substituted with Ns. Gubbins was used to remove recombination from all alignments. SNPs were identified using SNP-sites [16].

Multi-locus sequence typing was performed as described by Page *et al*. [17]. Kleborate [18] was used to screen all *K. pneumoniae* isolates for AMR genes. Ariba and the ARG-ANNOT database (v2.0) were used for all other species [19]. Genes were considered present if they were found at more than 90% coverage and nucleotide identity. Plasmid incompatibility typing was performed using Ariba and PlasmidFinder (v2.1) [20].

Blood cultures collected within the hospital between 2016 and 2018 that were positive for *K. pneumoniae* were included in the sequence analysis to provide context to the outbreak and ICU data and to also identify any additional isolates related to the outbreak.

For *E. coli*, we had access to a national collection of clinical isolates’ sequence data, which was used to provide context for the positive samples collected during the study period. These isolates were collected from within the UK between 2001 and 2018.

### Nanopore sequencing of outbreak isolates

DNA from a subset of outbreak isolates was extracted for nanopore sequencing (Table S1). Three isolates with long reads only underwent error correction using Nanopolish (https://github.com/jts/nanopolish). Four isolates with short-read data underwent hybrid assembly using Unicycler with default settings [21]. Comparisons of *bla*_NDM-1_ carrying contigs were performed using Artemis and the Artemis Comparison Tool on ICP001, ICP153 and ICP181 [22].

### Statistical methods

Comparisons between categorical variables were made using chi-square tests (where six or more data points were available) or Fisher’s exact test (five or fewer data points). Distributions of continuous variables were compared using paired or unpaired two-sample two-tailed *t*-tests for normally distributed data and Wilcoxon–Mann–Whitney tests for non-normally distributed data.

### Ethics statement

The study protocol was approved by the NHS Health Research Authority, East of England - Cambridge Central Research Ethics Committee (REC ref: 15/EE/0318) and the Cambridge University Hospitals NHS Foundation Trust Research and Development department (ref: A093680).

## Results

**Fig. 3.**
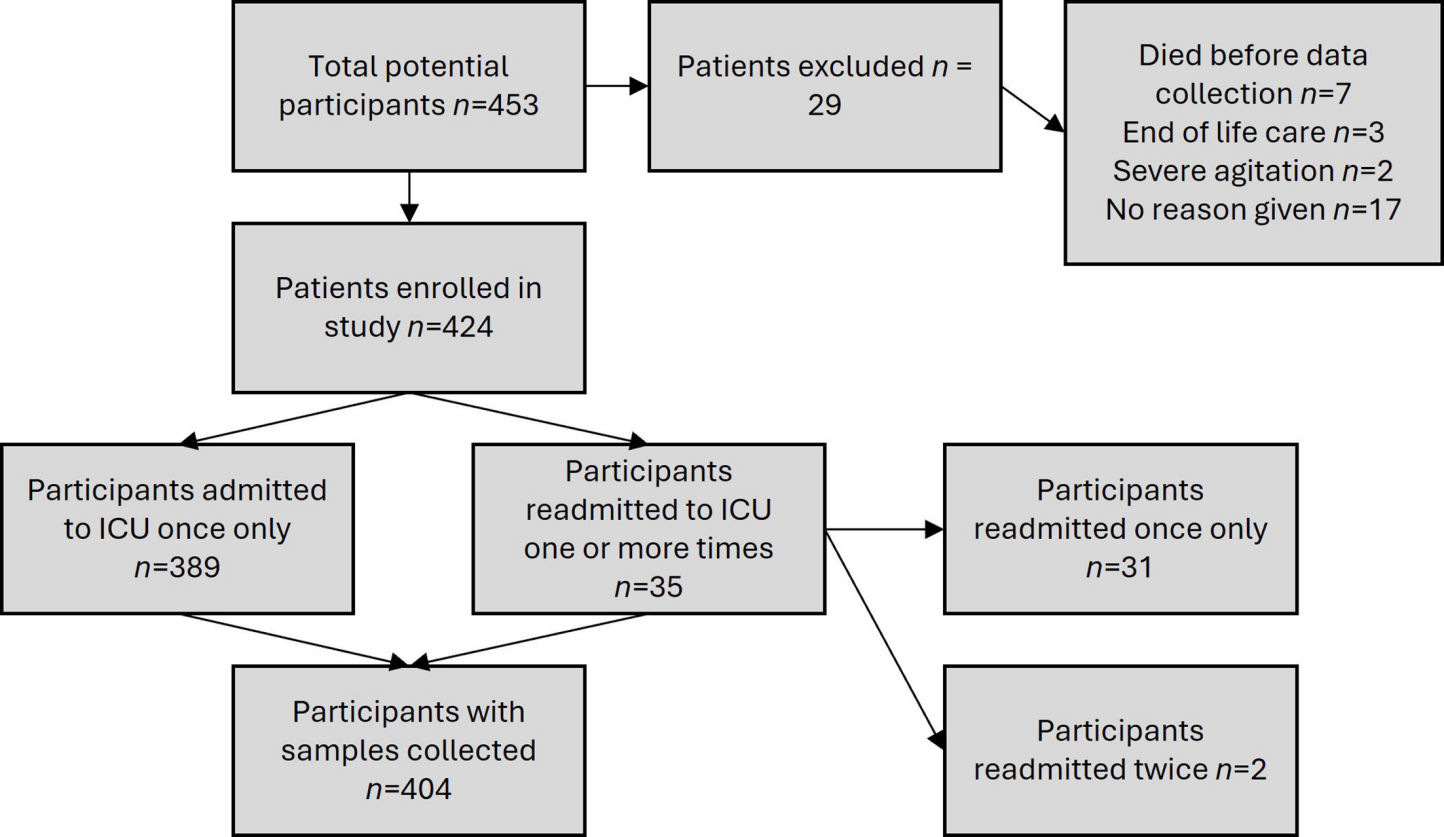
Flow diagram detailing the enrolment and exclusion of patients in the study.

Between June and December 2016, 453 ICU patients were potentially eligible for enrolment in the study. Of these, 29 participants were enrolled but not sampled due to the following reasons: death before data collection (*n*=7), end-of-life care (*n*=3), severe agitation (*n*=2) and no reason recorded (*n*=17), leaving 424 patients to sample across the study period. Thirty-five were readmitted once, of whom four were readmitted on two further occasions. See Fig. 3 for the flow diagram showing patient enrolment. Baseline characteristics of participants are summarized in Data S2 and S3. The median patient age was 61 years (range 44 to 74 years) and 242 (57%) were male. Carrier versus infection status was determined based on microbiology results and/or the clinical decision to treat patients for infection. The median admission length was 3 days [interquartile range (IQR) 1 to 6 days] in patients with no evidence of MDR GNB carriage or infection, whereas median admission length was significantly longer in those carrying or infected with MDR GNB at 6 days (IQR 2 to 12 days, *P*<0.0001, Wilcoxon–Mann–Whitney test). Patients with previous ICU admissions were more likely to be carriers of/infected with MDR GNB (*P*<0.0001, chi-square test).

### Carriage or infection with MDR GNB during the study period

A total of 404 patients had screening samples collected on their first ICU admission, and 59 (15%) were positive for at least 1 target MDR GNB, of which 16 patients were positive for multiple MDR GNB: 2 species (*n*=14), 3 species (*n*=1) and 4 species (*n*=1), respectively. *P. aeruginosa* was identified most frequently (*n*=24), followed by *E. coli* (*n*=14) and *K. pneumoniae* (*n*=9) (Table S1), and our genomic analyses therefore focus on these three species. A breakdown of positive samples per site can be found in Data S4.

Nine MDR GNB-positive blood samples were identified from six different patients who were part of the study, including the outbreak investigation.

Of the three most frequently identified species (*E. coli, K. pneumoniae* and *P. aeruginosa*), most positive samples were those associated with stool (rectal swabs and faecal samples). In the case of *E. coli*, 73% (*n*=24/33) of positive samples were stool associated. For *K. pneumoniae*, 60% (*n*=36/60) of positive samples were stool associated, and in *P. aeruginosa*, this was 46% (*n*=58/127). As expected, *P. aeruginosa* was the most frequently identified pathogen in respiratory samples (tracheal aspirates and sputum). In total, 47% (*n*=60/127) of samples positive for *P. aeruginosa* were found in these samples compared to 12% of samples positive for *E. coli* being respiratory associated and 15% (*n*=9/60) for *K. pneumoniae*.

Of those sampled on their first admission, 49.5% (200/404) had a full set of samples collected. In this calculation, a full set was one of each stool, urine and respiratory samples. Wound samples were excluded as not all patients presented with a wound. A total of 50.5% of patients sampled at admission did not have a full set collected. Of this, 80.4% (164/204) had two samples collected, and 19.6% (40/204) had one sample collected. The median number of samples collected across all first admissions was 2 (IQR 2–3).

Most patients (345/404, 85.4%) were negative for MDR GNB during their first admission to ICU. Following previously negative samples, 30/345 patients (8.7%) subsequently tested positive for an MDR GNB during their ICU stay. Phylogenetic links between patients who acquired MDR GNB during the study are described by species in the sections below. The median time to acquisition of MDR GNB was 8 days (range 1 to 136 days, IQR 4.3 to 20.8). *P. aeruginosa* was the most frequently acquired species (*n*=16), followed by *E. coli* (*n*=7) and *K. pneumoniae* (*n*=4). In screening samples, *P. aeruginosa* was most frequently identified in stool samples following previously negative samples. In the case of *K. pneumoniae*, the pathogen was found in all sample types (wounds, respiratory, urine and stool) that were previously negative. *E. coli* was most frequently found in stool samples following previously negative results. Following previously negative samples, three patients tested positive for multiple pathogens. Patient ICU084, an immunosuppressed patient with an extended ICU stay, was positive for *K. pneumoniae* and *P. aeruginosa* following readmission from the transplant unit. They acquired *Elizabethkingia meningoseptica* and *Stenotrophomonas maltophilia* 39 and 91 days later, respectively. Following negative admission samples, ICU090 had positive discharge samples for *Citrobacter freundii* and *E. coli*, and patient ICU099 acquired *E. coli* and *K. pneumoniae* after 15 days.

In the context of clinical samples, four blood samples were collected from ICU336, which were positive for *E. coli*. Five blood samples were positive for *K. pneumoniae*. Three samples were collected from IDA0002, one from NCCU001 and one from L4001.

### Investigation of an undetected *K. pneumoniae* outbreak

**Fig. 4.**
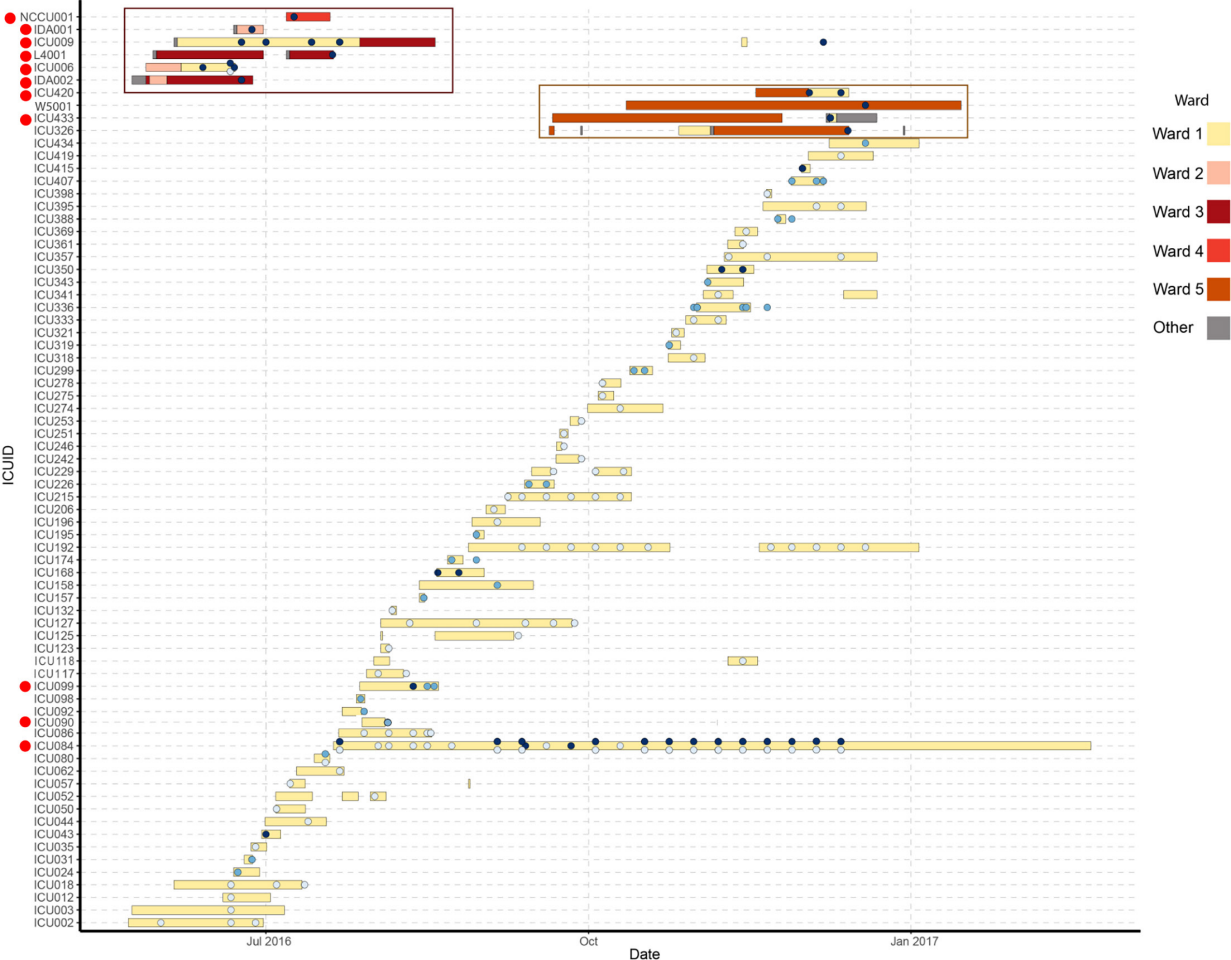
Timeline demonstrating all patients positive for *P. aeruginosa,* MDR *E. coli* or *K. pneumoniae*. Horizontal bars represent individual patient admissions, coloured according to hospital ward. Filled shapes indicate positive samples. Light blue indicates *P. aeruginosa,* mid-blue *E. coli* and dark blue *K. pneumoniae*. The *K. pneumoniae* outbreaks are highlighted in red and orange boxes. Ward 1 was the ICU. Specific patients discussed in the context of this figure have been highlighted with red circles. Seventy-three patients are included in this figure, consisting of all patients positive for one or more *P. aeruginosa*, *E. coli* and/or *K. pneumoniae*. Also included are patients involved in the *K. pneumoniae* outbreak but not enrolled in the ICU study.

During the first week of the study, two patients (ICU006 and ICU009) tested positive in their faecal samples for CR *K. pneumoniae*. A separate CR *K. pneumoniae* bacteraemia had been identified on ward 2. Enhanced surveillance of wards linked by admission to ICU006 and ICU009 identified four further carriers [IDA001 (ward 2), L4001 and IDA002 (ward 3) and NCCU001 (ward 4)], over 36 days (Fig. 4, red box). IDA002 was initially positive in a blood sample, followed by positive rectal swabs 48 h later; however, rectal swabs were not collected until they were linked to the outbreak. Both NCCU001 and L4001 were positive for the suspected outbreak strain in blood samples collected ~3 weeks after we identified ICU006 and ICU009 in our study. IDA001 was positive for the potential outbreak strain from a rectal swab. Enhanced screening consisted of weekly carbapenemase-producing *Enterobacterales* (CPE) screening samples collected from all patients linked to the outbreak by admission and all patients on the ICU. No further cases were identified for 5 months, until two patients (ICU420 and ICU433) tested positive for CR *K. pneumoniae* from rectal swabs. Both patients previously stayed together on ward 5 in the same eight-bedded bay. Ward 5 screening identified two further carriers, all with close ward contact (Fig. 4, orange box). In total, 10 patients tested positive at any site for the outbreak strain. Three environmental sites on ward 5 (two sluices and one sink in the eight-bedded bay) were positive for CR *K. pneumoniae*. The sink in the bay was found to be positive after the positive patients had been moved from the bay and after routine cleaning. The sluice was used by all areas of the ward for waste disposal. All isolates were resistant to all antibiotics tested except colistin (Table S1). PCR assay suggested the presence of a metallo-beta-lactamase, which was confirmed by WGS as *bla*_NDM-1_.

**Fig. 5.**
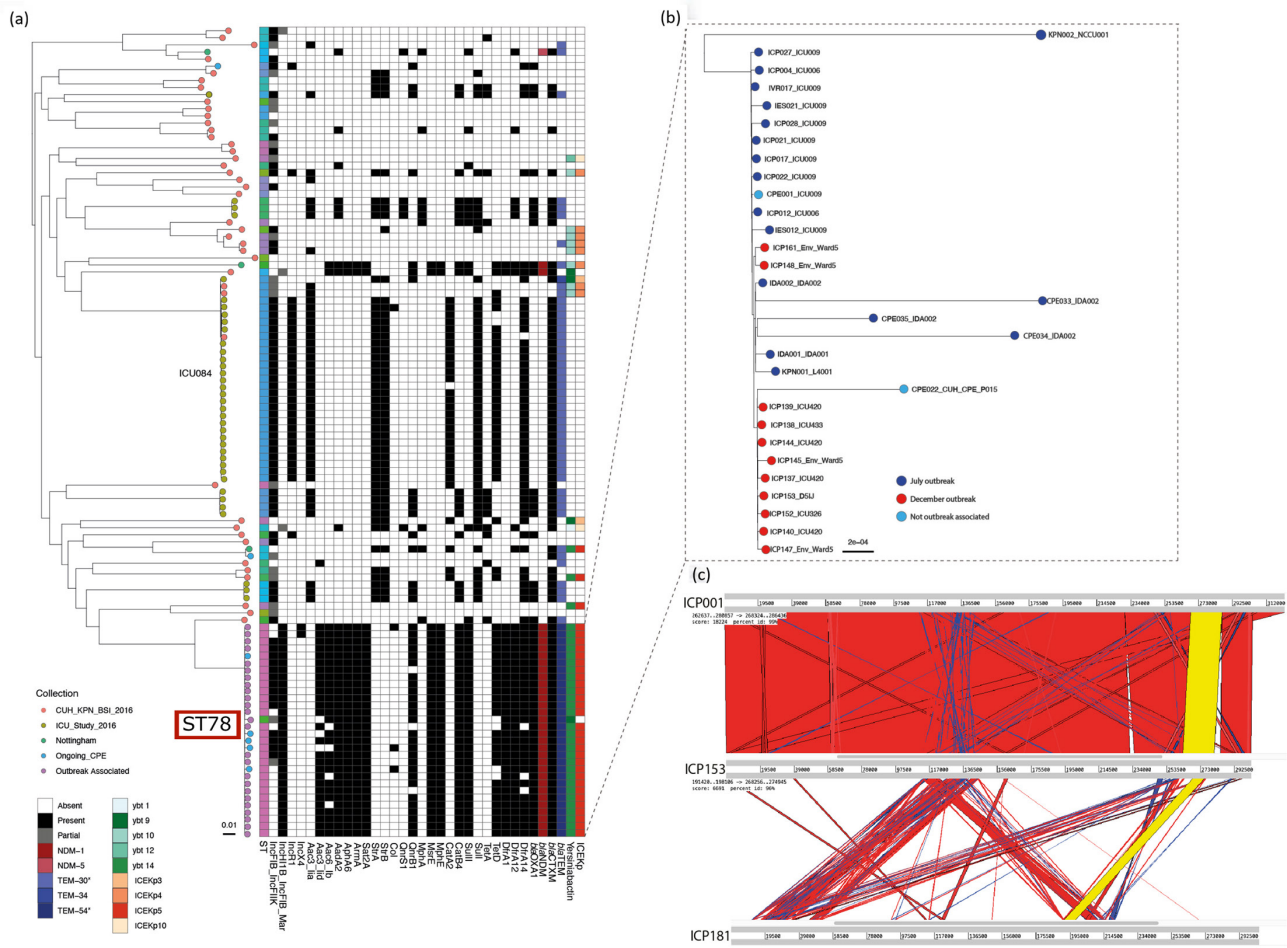
Core SNP phylogeny of *K. pneumoniae* isolates. (a) Core genome SNP phylogeny of outbreak and contextual isolates. Terminal branches denote isolate collection, detailed bottom left. Outbreak isolates are depicted by a red box showing ST78. The second-largest cluster of isolates originates from ICU084 and is named for clarity. Gene presence and absence are depicted in the adjacent grid where a filled box indicates gene presence and white indicates absence of the gene. (b) Enlarged view of outbreak isolates. Dark blue tips show the June/July outbreak, red December outbreak and light blue isolates were part of the cluster but not epidemiologically linked to outbreaks. (c) Artemis Comparison Tool (ACT) comparison between three *bla*_NDM-1_-positive isolates. Regions displayed are contigs produced by long-read sequencing containing the *bla*_NDM-1_ gene and the associated plasmid. The yellow bar depicts the *bla*_NDM-1_ matching region between the three isolates. Red bars show matching regions. Blue bars show matching but inverted regions.

To examine the outbreak further, we investigated additional *K. pneumoniae* bacteraemia isolates and any CR *K. pneumoniae* samples collected between January 2016 and December 2018. A phylogeny based on core genome SNPs showed typical deep-branching *K. pneumoniae* lineages*,* except for a group of highly related isolates originating from the outbreak and ward 5 environmental samples (Fig. 5). In contrast, six study participants yielded different carbapenemase-negative, ESBL-positive *K. pneumoniae* lineages, with no detectable evidence of transmission.

All outbreak isolates were ST78, a single-locus variant of ST14 (Fig. 5). *K. pneumoniae* bacteraemia isolates from 2016 and CR *K. pneumoniae* from 2016 to 2018 at CUH were distributed throughout the tree. Kleborate identified an increase in acquired AMR genes in outbreak isolates (Fig. 5a). The minimum pairwise core genome SNP difference between outbreak and contemporary non-CR isolates was 13,128, whilst pairwise differences between outbreak isolates ranged from 0 to 19. Ward 5 environmental isolates were highly related to both June (2 to 16 SNPs) and December (1 to 10 SNPs) outbreaks. Sixty-two samples were collected from ICU084 over 144 days comprising 22 separate sampling sets. These samples differed by 0 to 8 SNPs (median 2) across multiple body sites, whereas the pairwise SNP difference between non-outbreak-associated patients was 79–23,558.

Sporadic cases of *K. pneumoniae* carrying *bla*_NDM-1_ were identified throughout the hospital after the outbreaks. Five isolates clustered with the outbreak (Fig. 5b), three of which originated from IDA002 in 2017. The isolates clustered with the patient’s original isolate but varied by at least 30 SNPs, suggesting diversification during prolonged patient carriage or diversification within an unidentified reservoir, which may have been human or environmental. The fifth isolate (CPE022) belonged to a non-study patient with multiple admissions to ward 5 during 2016, including 2 days in the outbreak bed bay and direct contact with ICU420 and ICU433.

### *bla*_NDM-1_ plasmid analysis of outbreak isolates

The highly mobile *bla*_NDM-*1*_ gene is frequently identified on various plasmids. We used long-read sequencing and hybrid assemblies to further characterize the *bla*_NDM-1_-positive isolates. Outbreak isolates carried IncHI1B and IncFIB_Mar replicons, corresponding to the dual-replicon plasmid IncH pNDM-MAR (JN420336) (Fig. 5a), most closely related to the CR PittNDM01 plasmid 1 (CP006799). We compared the June outbreak lineage *bla*_NDM-1_ region (ICP001) to one from the December outbreak (ICP153) and to a *bla*_NDM-1_-positive *K. pneumoniae* (ICP181) carried by ICU118 after the study period. High similarity was identified between ICP001 and ICP153 (Fig. 5c). In contrast, the ICP181 *bla*_NDM-1_ gene region corresponded to an IncF plasmid, showing multiple *bla*_NDM-1_ plasmids circulating in the hospital. Three ST405 *E. coli* isolates carried a *bla*_NDM-1_-positive IncA/C plasmid, and an *Enterobacter cloacae* isolate carried an IncHI2A plasmid, although these were identified from short-read data only.

Enhanced screening and infection control protocols were instituted on the ICU and epidemiologically linked hospital wards following detection of the outbreak, and additional cases were not detected, although sporadic cases of *bla*_NDM-1_-positive isolates continued to be identified. These protocols included screening patients in the same bay or clinical areas as index cases, enhanced bleach cleaning and hydrogen peroxide vapour decontamination of side rooms, bleach and ultraviolet C cleaning of sluices and the addition of extra cleaners for high-risk touch points. Any new positive patient screens triggered an incident management meeting where the closure of wards or bays to facilitate cleaning was considered.

### *K. pneumoniae* environmental and patient transmission

Potential spread of ESBL-positive *K. pneumoniae* between patients and the environment was identified on one occasion outside of the outbreak (Data S5). ICU099 carried the same lineage in their stool as environmental samples collected from their room. The two isolates differed by 0 SNPs, highlighting potential transmission opportunities for the strain. Re-sampling of this area in the next month as part of the protocol did not identify this strain, suggesting that cleaning and disinfection following patient discharge was successful. ICU084 also carried a lineage with only 2 SNP differences compared to an isolate identified in their immediate environment. The locations of positive environmental samples within the ICU are shown in Data S1.

### *P. aeruginosa* was the most frequently identified MDR GNB

*P. aeruginosa* was identified in 42/408 (10%) study patients. Sixty isolates originated from the respiratory tract, 59 from the gastrointestinal tract, 8 from urine and 3 from wound swabs. Fourteen isolates were removed due to contamination or quality control issues, leaving data from 35 patients. As expected, no ESBL genes were identified in any of the *P. aeruginosa* isolates. One respiratory isolate from ICU0080 carried a *bla*_IMP-26_ gene, an additional aminoglycoside resistance gene (*aac6*), *catB3*, which is associated with chloramphenicol resistance, and the trimethoprim resistance gene *dfrA1*, which were not identified in other isolates.

### Sequence typing and SNPs untangle transmission

**Fig. 6.**
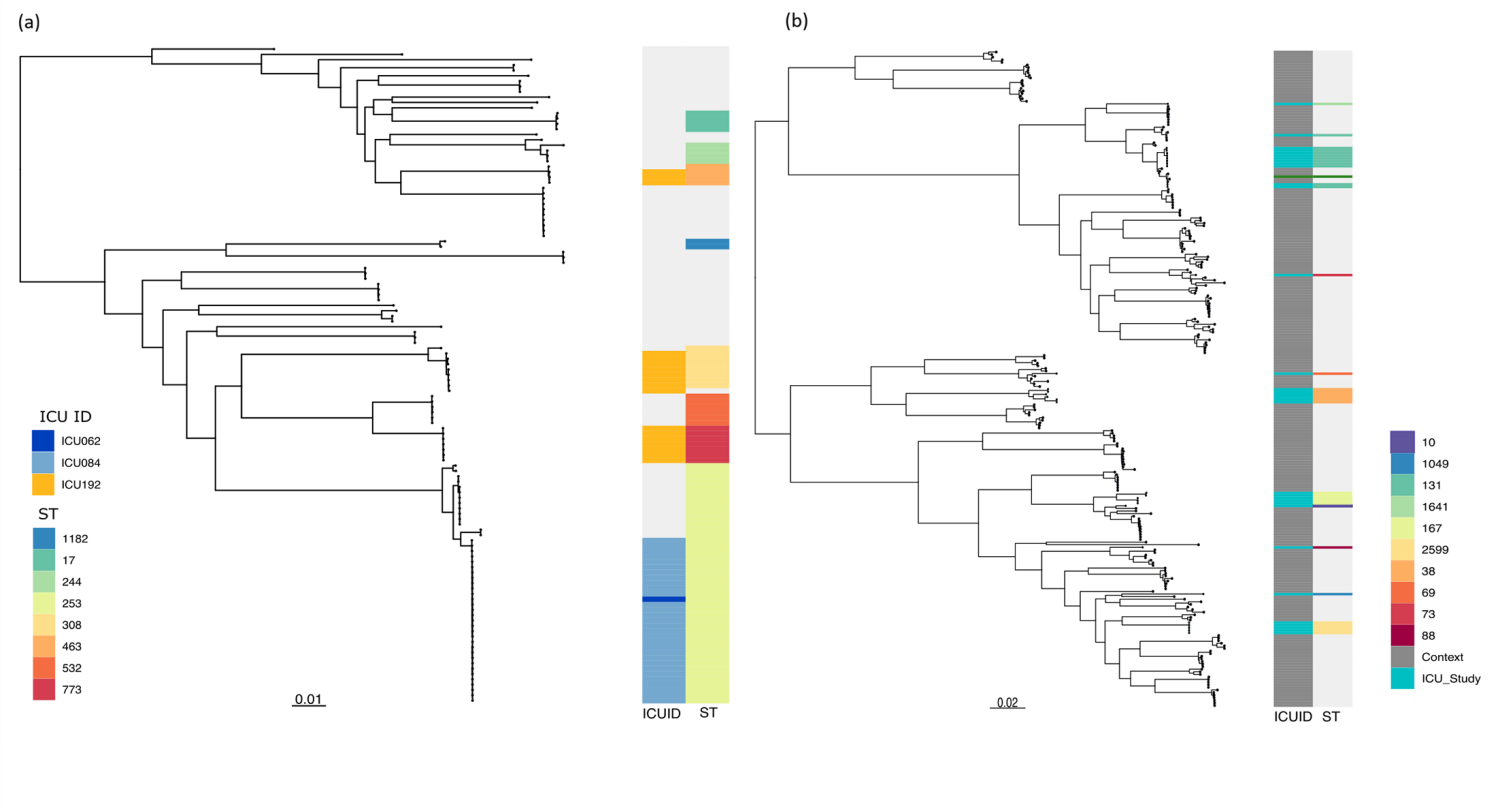
Core SNP phylogeny of *P. aeruginosa* isolates and *E. coli* collected during the study period.(a) Core SNP phylogeny of *P. aeruginosa* isolates. The close relationship between ICU084 and ICU062 and the different STs carried by ICU192 are highlighted in orange and shown in the left-hand column ‘ICUID’. STs shared by multiple patients only are shown in the right-hand ‘ST’ column. (b) Core SNP phylogeny of *E. coli* isolates. UK context isolates are shown in grey in the left-hand column, ‘ICUID’, and ICU study isolates are in turquoise. STs shared by multiple patients only are shown in the right-hand column ‘ST’. ST10 found in ICU098, ST69 found in ICU343, ST73 found in ICU158, ST88 found in ICU092, ST1049 found in ICU080, ST1641 found in ICU158 and ST2599 found in ICU336.

Thirty STs were identified, and six contained isolates from multiple patients: ST17, ST244, ST463, ST532 and ST1182 (Fig. 6a). Twenty patients carried STs not identified in other sampled patients. The largest cluster, ST253, was identified in 7 patients and the minimum pairwise difference between patients was 7 and the maximum 5,201. There were 7 to 27 SNPs between isolates from ICU062 and ICU084. Both patients resided on the ICU at the same time (Fig. 4); however, the level of sampling prevents implication of direct transmission between patients. Isolates collected from ICU057 and ICU086 had 45 to 50 SNPs between them. Whilst the two carried the same lineage, direct transmission was unlikely, and the two patients stayed on the ICU 1 week apart. ICU086 was positive for this lineage across all four sample sites (stool, respiratory, urine and wound), and ICU057 was positive in rectal and sputum samples. Outside of these four patients, SNP differences between patients were a minimum of 717, demonstrating carriage of different lineages within the ST. ICU084 contributed 31 ST253 isolates, collected from respiratory, gastrointestinal and urinary samples. There were 4 to 35 SNPs between respiratory isolates and 11 to 30 SNPs between the gastrointestinal isolates. Only one urine sample contained *P. aeruginosa*. When comparing sample sources, there were 6 to 38 SNPs between respiratory and gastrointestinal isolates, supporting within-host diversity. The urine sample differed by 14 SNPs from the other sources (Figs 1 and 3).

ICU192 and ICU274 both carried ST463 and spent 21 days in the ICU together. ICU192 was positive for this ST in both stool and respiratory samples, whilst ICU274 was positive in stool only. ICU274 was negative on admission and then subsequently positive for the same *P. aeruginosa* ST as ICU192 after 1 week. Despite carrying the same ST and being on the ward together, their isolates differed by 125–144 SNPs. ICU192 was a complex patient with multiple comorbidities and admissions during the study. Three STs, ST463 which had a within-ST median SNP difference of 52, ST773 (within-ST median SNP difference of 28) and ST308 (within-ST median SNP difference of 80), were identified at different times in ICU192’s respiratory and stool samples. Between-ST SNP differences ranged from 346 to 55,142, demonstrating carriage of multiple lineages simultaneously, although ST308 was detected only during ICU192’s second admission to ICU.

*P. aeruginosa* was identified in the environment on three occasions. In one case, ICU192 carried in their stool and sputum the same ST as an isolate collected from taps in one of the ward side rooms. This patient had not stayed in this room during the study. Similarly, isolates from ICU127 were the same ST as an isolate collected from inside a tap. We did not, however, identify any opportunities for direct transmission. The locations of positive environmental samples within the ICU are shown in Data S1.

### ESBL-producing *E. coli* populations showed low transmission between ICU patients

A total of 33 ESBL-producing *E. coli* (ESBL-Ec) samples were identified in 19/408 (5%) patients sampled during the study (Fig. 4), and the majority were found in the gastrointestinal tract. All positive patients were ESBL-Ec carriers, except ICU336 who also had ESBL-Ec bacteraemia. No ESBL-Ec were identified in environmental samples. *bla*_CTX-M-15_ was detected in 21/33 (63%) isolates and *bla*_CTX-M-14_ in 5/33 (15%). Three isolates displayed phenotypic susceptibility to cefotaxime. The remaining four showed phenotypic ESBL characteristics, suggesting possible plasmid loss between the phenotypic and genotypic testing. Asymptomatic carriage of a *bla*_NDM-1_-positive *E. coli* was identified in one patient admitted from a different hospital with a history of foreign travel.

### Multiple ESBL-Ec lineages were identified amongst study participants

A total of 224 isolates consisting of 219 UK isolates spanning 2001–2018, selected from a curated collection [15], and 5 isolates collected as part of CPE surveillance at CUH were added to provide context to ICU data. Study isolates were dispersed throughout the tree, demonstrating carriage of multiple ESBL-Ec lineages amongst patients (Fig. 6b). Ten different STs were identified, with ST131 being the most frequent (11/33 isolates, 33%, 5/19 patients, 26%). ST38, ST131 and ST167 were shared amongst multiple patients, eight patients carried unique STs and two STs were identified in ICU336 (Fig. 6b). ICU336 had ST131 isolated in two blood samples and ST2599 in five subsequent samples (blood *n*=2 and stool *n*=3), demonstrating within-host carriage of matching and varied lineages across multiple body sites. Of the seven patients that acquired ESBL-Ec, there were two instances where patients acquired the same ST.

Ten of the 11 ST131 isolates clustered together in the C2/H30Rx clade, and interpatient diversity ranged from 70 to 635 SNPs. Six isolates from ICU336 (blood *n*=2) and ICU407 (stool *n*=2 and urine *n*=2) had 0 SNP differences between them, but patient movement data identified no direct contact (Data S5).

ST167, which is part of the larger ST10 complex, was identified in the stool of four study patients (ICU024, ICU031, ICU099 and ICU319) associated with hepatology. Samples from ICU099 and ICU319 differed by only 11 SNPs, yet the patients were on the ICU 2 months apart. By auditing expanded patient movement data, we identified potential transmission between these two patients on a hepatology ward, where both were patients in the same bed at different times within 24 h. In the case of ICU024 and ICU031, there were 122 SNPs between their isolates. Pairwise comparison outside of these two instances showed that the isolates between patients differed by more than 10,000 SNPs. Sampling depth precludes further inference on transmission, and using ST as evidence for transmission is complex due to varying levels of within-ST diversity.

## Discussion

ICU patients are disproportionately affected by MDR GNB, resulting in increased morbidity and mortality, but the true burden of MDR GNB in critical care settings is incompletely understood [23,24]. To investigate, we performed a longitudinal genomic surveillance study to determine the rate of carriage, infection and transmission of MDR pathogens in an adult ICU in the UK.

The MDR GNB asymptomatic carriage rate was 15% on admission, and an acquisition rate of 8.7% was identified, which correlated with similar populations. However, considerable variations in study designs complicate comparisons between reported rates [24,28].

Prospective surveillance identified a multi-ward outbreak of CR *K. pneumoniae*, resulting in enhanced CRE screening and infection control procedures across epidemiologically linked wards. The absence of cases on the ICU between July and December 2016 suggests the outbreak potentially persisted unsampled elsewhere in the hospital. This was the first *bla*_NDM-1_ outbreak in our hospital following a single previous, unrelated case in 2010. The index case had no risk factors for CRE and would not have been eligible for CRE screening according to NHS screening protocols at the time of the study. The first samples positive for the outbreak strain from this patient were rectal screening swabs. Sputum and tracheal aspirates were negative for the first two sampling periods. Following these negative samples, a third respiratory sample was positive for the outbreak strain. These respiratory samples were collected as part of the study protocol. The outbreak strain would not have been detected unless clinical management required the collection of respiratory samples.

Although asymptomatic carriage, fomites and healthcare workers are potential sources of transmission, most positive patients carried discrete lineages, supporting the effectiveness of the routine ICU IPC protocols used at the time [9,29, 30]. The same STs in *P. aeruginosa* and *E. coli* were identified in multiple patients. The majority of ST131 *E. coli* belonged to the C2/H30Rx clade [31], but inter-patient isolates differed by more than 70 SNPs. This indicates patients carried *E. coli* lineages circulating in the wider population, rather than lineage transmission within the ICU [32]. We identified the same ESBL-Ec lineages in two patients, yet found no evidence of direct contact, which may represent part of a transmission chain. Combined epidemiological and genomic analysis at the SNP level of *P. aeruginosa* lineages provided increased clarity when identifying transmission rather than relying on shared STs. Sharing of ST167 by hepatology patients initially suggested that we had identified a discrete lineage circulating in this group. However, deeper analysis using SNP data supported transmission between two of the four patients only. We uncovered an unidentified burden of MDR GNB in an ICU, but to completely elucidate transmission networks in the hospital, the study would need to include ICU staff screening, more frequent environmental sampling on the ICU and be extended to include non-ICU wards. The discovery of an outbreak of CR *K. pneumoniae* that had spread silently to several wards provides support for this.

Longitudinal sampling across multiple body sites provides an improved understanding of the dynamics of MDR pathogen dynamics [7,33, 34]. We identified co-colonization with ST131 at different body sites, which is associated with progression to infection and carriage of multiple STs in ICU192 [35]. Multiple colony sequencing would increase clarity regarding lineage distribution but was prohibited by funding constraints.

We acknowledge several limitations to our study. The study was conducted only in an adult ICU, except when additional sampling outside the ICU was directed by the IPC Lead during the outbreak. Therefore, the results may not be generalizable to other settings. The inclusion of other wards, such as those that commonly transfer patients to and from ICU, would have provided a broader understanding of MDR GNB in high-risk patients. Although we aimed to enrol all patients admitted to ICU during the study period, this was not always possible. Reasons for exclusion included patient refusal, staffing pressures or end-of-life care. Incomplete sampling of the study population may have affected the ability to identify all transmission events, and indeed, we identified genomic evidence of transmission where reliable epidemiological links could not be established. More intensive sampling of patients and staff members might have been helpful but was not feasible.

Increasing the frequency of environmental sampling to weekly rather than monthly might also have provided us with a more comprehensive picture of MDR GNB population structure within ICU and potential sources of transmission. However, this was not feasible during this study because of staffing and funding constraints.

This work has highlighted that NHS screening protocols for MDR GNB at the time were not adequate to identify patients at risk of carrying these organisms. The initial patients involved in the *K. pneumoniae* outbreak did not meet criteria for CPE screening. Therefore, our methodology of prospectively screening all patients admitted to ICU not only detected asymptomatic carriage of a highly antibiotic-resistant *K. pneumoniae* strain in patients but also enabled action by the IPC team to manage the outbreak. It is not possible to quantify the number of transmission events averted. However, knowledge of asymptomatic carriage identified patients to be isolated to reduce onward transmission. This enabled us to detect and respond to the outbreak more rapidly than would have been possible if we had relied on detection via clinically directed sampling. These findings suggest that screening for MDR GNB should be considered for all patients admitted to critical care settings and potentially extended to other high-risk wards (e.g. transplant wards). This would, however, have considerable practical and financial implications for the NHS. Whilst this brings additional costs, the genomic data produced has multiple opportunities for use. Tracking of AMR genes would provide further benefit to both patients and clinicians by supporting antimicrobial stewardship. A more rapid turnaround time also has the potential to reduce the need for empirical prescribing and would result in an in-depth data source of pathogen genomic data for use in further research.

We also did not screen ICU staff for MDR GNB during the study, as this was not routine clinical practice in the UK and there are no decolonization recommendations for colonized staff. Patients with MDR GNB were rapidly isolated and the limited number of staff in contact with them wore full personal protective equipment. Staff screening might have been considered by the IPC Team had the outbreak not been controlled relatively rapidly.

Our study demonstrates the value of adding genomic data to standard epidemiological methods. It adds detail and clarity to complex patient-pathogen relationships that can assist IPC Teams to delineate transmission events and potentially inform outbreak management (but only if performed in real time). However, this work also demonstrates that conducting combined epidemiological and genomic surveillance requires considerable additional resources (staff, time and skills) that may not be available outside a research setting in the routine healthcare system. Whilst it might have been interesting to perform conjugation experiments to confirm transfer of plasmids between different organisms, this was not done at the time of the study. A subsequent study conducted at our hospital has used long-read sequencing to examine genomic diversity and associated plasmid movement of carbapenemase-producing bacteria and determined that ~30% of isolates with carbapenemase genes on plasmids had acquired them via horizontal transmission [36].

Prospective screening of ICU patients for MDR GNB rapidly identified, and assisted in controlling, an outbreak of CR *K. pneumoniae*. WGS data built a picture of pathogen populations showing that outside of the outbreak, most patients carried discrete lineages representing circulating hospital and community MDR GNB populations, which has also been identified in other ICUs in the UK [24]. As a result of this study, all patients admitted to critical care units at CUH are routinely screened for CRE, and those with pre-existing risk factors for CRE are isolated until they have three negative swabs. The IPC team elected to instigate this given that the patients involved in the outbreak would not have been screened under the guidelines in place at the time. Our findings support the use of standard infection control protocols for patients admitted to hospital and CPE screening in ICU settings. Furthermore, rapid WGS can be beneficial for investigation of outbreaks caused by MDR GNB and should be considered if feasible to implement in local settings.

## Supplementary material

10.1099/mgen.0.001654 Uncited Supplementary Material 1.

10.1099/mgen.0.001654 Uncited Supplementary Material 2.

## Abbreviations

CPE: carbapenemase-producing *Enterobacterales*
CR: carbapenem resistance
CRE: carbapenem-resistant *Enterobacterales*
CUH: Cambridge University Hospitals NHS Foundation Trust
ESBL: extended-spectrum beta-lactamase
ESBL-E: Extended-spectrum beta-lactamase-producing *Enterobacterales*
ESBL-Ec: ESBL-producing *Escherichia coli*
GNB: Gram-negative bacteria
HCAI: healthcare-associated infection
ICU: intensive care unit
IPC: Infection Prevention Control Team
IQR: interquartile range
MDR: multidrug resistance
MDR GNB: multidrug-resistant Gram-negative bacteria
ST: sequence type
WGS: whole-genome sequencing
