# Supplementary material for: Whole-genome sequencing to investigate the prevalence and transmission of multidrug-resistant Gram-negative pathogens in an adult intensive care unit in the UK
Source: Microb Genom. 2026 May 6;12(5):001654. doi: 10.1099/mgen.0.001654 (PMC13148723; doi:10.1099/mgen.0.001654)
Supplement: Uncited Supplementary Material 1. [file mgen-12-01654-s001.pdf]

ICU floor plan

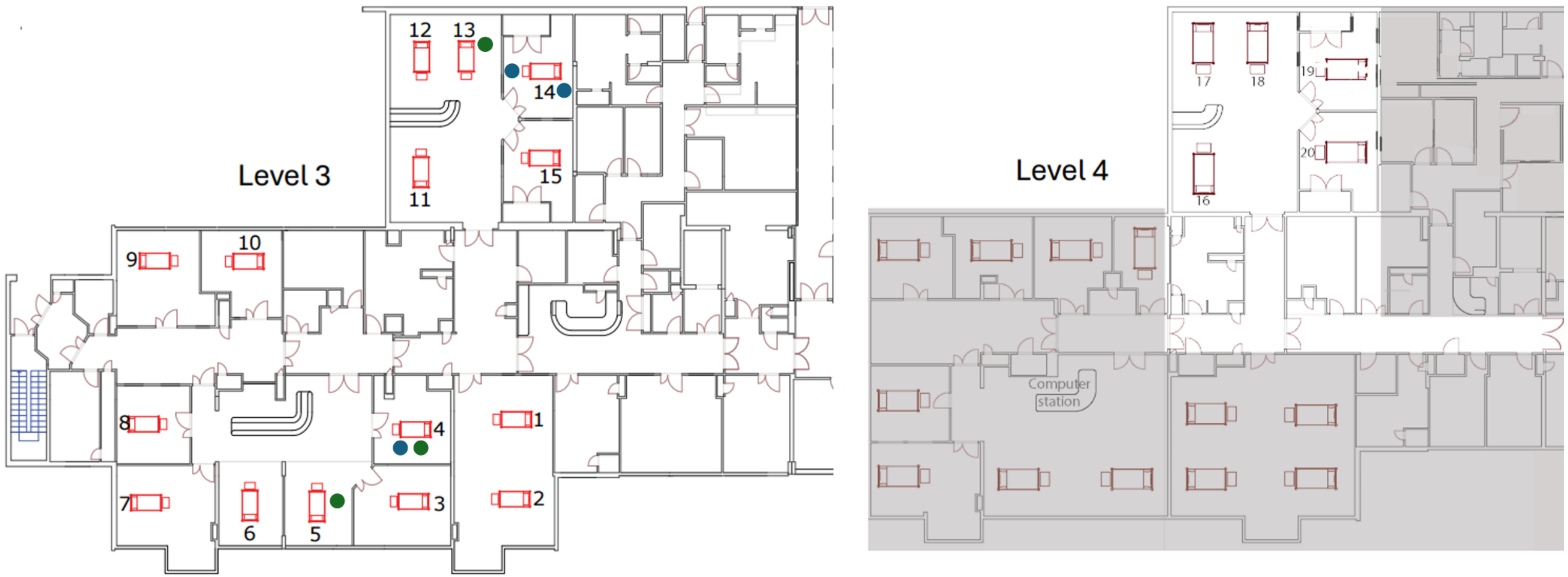

Supplementary Data S1: Floorplan of ICU unit on Level 3 and 4. Grey area is a ward unrelated to the ICU. The locations of positive environmental sites are shown by blue (*K. pneumoniae)* and green circles (*E. coli*).

Characteristic		None (n=269)	Carrier/Infection (n=155)	*p* value
Gender (female)		127 (47.2%)	55 (35.5%)	0.019
Age (yrs)(mean, median (IQR))		57.9, 61 (44-73)	59.7, 61 (48-74)	0.32
Length of stay (days)(mean, median (IQR))		5.1, 3 (1-6)	10.8, 6 (2-12)	<0.001
Prev ICU admission (n=423)		30 (11.2%)	37 (23.9%)	0.001
Previous ADH admission (n=423)		104 (38.8%)	79 (51.0%)	0.015
Admitted from (n=422)	Own home<br>Other hospital<br>Long term care	206 (77.2%)<br>55 (20.6%)<br>6 (2.3%)	106 (68.4%)<br>46 (29.7%)<br>3 (1.9%)	0.11
Previous colonisation	MRSA (n=421)	10 (3.8%)	11 (7.1%)	0.12
	CDI (n=393)	1 (0.4%)	3 (2.1%)	0.15
	VRE (n=386)	4 (1.6%)	15 (10.6%)	<0.001
	ESBL (n=385)	7 (2.9%)	8 (5.7%)	0.17
	CPE (n=408)	1 (0.4%)	4 (2.7%)	0.063
Antibiotics	Current (n=423)	232 (86.6%)	142 (91.6%)	0.12
	Last 30 days (n=356)	158 (72.8%)	125 (89.9%)	<0.001

Supplementary Data S2: Summary of basic demographics of enrolled patients.

Characteristic		None (n=269)	Carrier/Infection (n=155)	*p* value
Co-morbidities	Diabetes mellitus (n=422)	65 (24.3%)	30 (19.4%)	0.24
	Immunosuppression (n=414)	60 (22.7%)	42 (28.0%)	0.23
	Haematological malignancy (n=421)	14 (5.3%)	6 (3.9%)	0.52
	Solid organ transplant (n=423)	36 (13.4%)	31 (20.0%)	0.15
	End stage renal failure (n=421)	20 (7.5%)	16 (10.4%)	0.59
	Cirrhosis (n=422)	33 (12.3%)	36 (23.4%)	0.003
	Respiratory disease (n=422)	83 (31.0%)	45 (29.2%)	0.71
	Cerebrovascular disease (n=420)	35 (13.2%)	18 (11.7%)	0.66
Presentations on admission**	Sepsis (n=421)	45 (16.9%)	34 (22.1%)	0.19
	Bacteraemia (n=423)	7 (2.6%)	4 (2.6%)	1.0
	Pneumonia (n=415)	52 (19.7%)	43 (28.5%)	0.041
	Line infection (n=419)	4 (1.5%)	4 (2.6%)	0.47
	Biliary (n=422)	5 (1.9%)	4 (2.6%)	0.73
	Acute pancreatitis (n=421)	4 (1.5%)	9 (5.8%)	0.019
	Peritonitis (n=418)	10 (3.8%)	8 (5.3%	0.48

	Infectious diarrhoea (n=420)	2 (0.75%)	4 (2.6%)	0.20
	Urinary tract infection (n=422)	9 (3.4%)	6 (3.9%)	0.79
	Skin and soft tissue infection (n=419)	7 (2.6%)	6 (4.0%)	0.45
Died during first admission		43 (16.0%)	17 (11.0%)	0.15

Supplementary Data S3: Summary of comorbidities and infections present on admission in study participants.

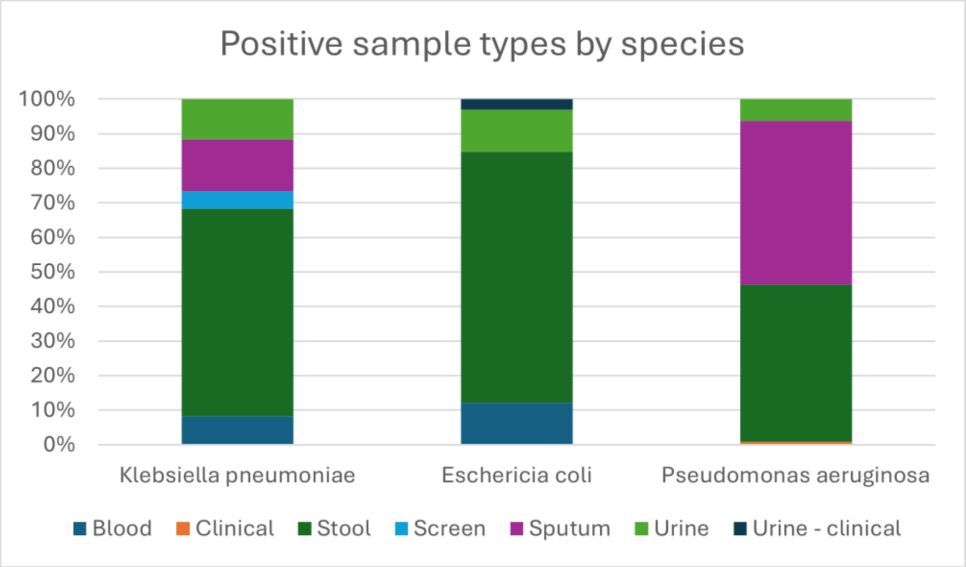

Supplementary Data S4: Positive sample types by species recovered from participants enrolled in the study. Rectal swabs and faeces samples have been amalgamated into 'Stool'. Tracheal aspirates and sputum samples have been amalgamated into 'Respiratory'. Urine – clinical – indicates a positive urine sample collected as part of routine clinical care rather than by the study team.

Species	Composition	Patients	ST	Genomic and epidemiological link	Type 1	Genomic but no epidemiological link	Type 2
K. pneumoniae	Study samples, CPE screening, Environmental, CPE surveillance	9	78	July: ICU006, ICU009, IDA001, L4001. December: ICU420, ICU433, ICU326, D5IJ, Environmental	July: ward overlap (ICU, Ward 2, ward 3) December: ward overlap (ICU and ward 5)	NCCU001	Isolate clusters with outbreak group
K. pneumoniae	Study samples, environmental sample	1	251	ICU099	Stayed in room with matching environmental isolate		
K. pneumoniae	Study samples, environmental sample	1	307	ICU084	Stayed in room with matching environmental isolate		
E. coli	Study samples	3	38	ICU226, ICU299	Ward overlap (ICU)	ICU434	Shared ST
E. coli	Study samples	5	131			ICU174 and ICU195. ICU336, ICU388 and ICU407	Shared ST
E. coli	Study samples	4	167	ICU024, ICU021	Ward overlap (ICU)	ICU099, ICU319	Shared ST
P. aeruginosa	Study samples	3	17	ICU044, ICU196, ICU333	No ward overlap, all stayed in bed 13 at different times		
P. aeruginosa	Study samples	3	244	ICU035, ICU117, ICU296	No ward overlap, all stayed in same bed bay at different times		
P. aeruginosa	Study samples	7	253	ICU050, ICU057, ICU062, ICU084, ICU086, ICU242, ICU398	Ward overlap (ICU)		
P. aeruginosa	Study samples, environmental sample	2	308	ICU003, environmental sample	ICU003 stayed in same location as environmental isolate	ICU003, ICU192	Shared ST
P. aeruginosa	Study samples	2	463	ICU192, ICU274	Ward overlap (ICU)		

P. aeruginosa	Study samples	5	532	ICU143, ICU246, ICU253	Ward overlap (ward 5)	ICU006, ICU395	Shared ST
P. aeruginosa	Study samples	2	1182	ICU318, ICU321	Ward overlap (ICU)		

Supplementary Data S5: Summary of shared STs. Composition: details the origin of isolates in each shared ST cluster. **Patients:** number of unique patients in each cluster. **ST:** sequence type of isolates in each cluster. **Genomic and epidemiological link:** details the patient and non-patient origins of isolates in each cluster that are genetically and epidemiologically linked. **Type 1:** details the type of epidemiological link between patients with genomically related isolates in the cluster. **Genomic but no epidemiological link:** details the patients in the cluster when no epidemiological link was identified. **Type 2:** details of the link between patients in the cluster where no epidemiological link has been found.
